# Prevalence of feline leukemia virus and feline immunodeficiency virus in cats from southern Italy: a 10-year cross-sectional study

**DOI:** 10.3389/fvets.2023.1260081

**Published:** 2023-11-06

**Authors:** Giovanna Fusco, Luisa Marati, Annamaria Pugliese, Martina Levante, Gianmarco Ferrara, Esterina de Carlo, Maria Grazia Amoroso, Serena Montagnaro

**Affiliations:** ^1^Unit of Virology, Department of Animal Health, Zooprofilactic Institute of Southern Italy, Portici, Italy; ^2^Department of Veterinary Medicine and Animal Production, University of Naples “Federico II”, Naples, Italy

**Keywords:** FIV, FeLV, feline retroviruses, cross-sectional study, southern Italy

## Abstract

**Introduction:**

Feline immunodeficiency virus (FIV) and feline leukemia virus (FeLV) are well-known retroviruses causing important infections in domestic cats worldwide. The goal of this study was to determine the prevalence of FeLV and FIV infections in cat living indoor and outdoor in southern Italy.

**Methods:**

The survey was conducted on 1322 stray and owned cats from the regions of Campania, Basilicata, and Calabria. It was carried out over a 10-year period to obtain a more realistic picture of the prevalence of these retroviral diseases in the country. FIV and FeLV status was determined by enzyme-linked immunosorbent assay (ELISA) using a commercial kit (SNAP Combo Plus FeLV/FIV, IDEXX). Risk factors were analysed by logistic regression.

**Results and Discussion:**

The results showed that 101/1322 (7.64%) cats were positive for FeLV antigen and 110/1322 (8.32%) cats were positive for FIV antibody. Twenty-six of the 1322 cats (1.97%) were positive for both FIV and FeLV infection. Our results are similar to those published in recent studies in Europe. A statistically significant association (*p* < 0.05) was found between year, province, region, lifestyle and risk of FeLV infection. FIV positivity was instead statistically associated only with year and lifestyle.

## 1. Introduction

Feline leukemia virus (FeLV) and feline immunodeficiency virus (FIV) are retroviruses that cause significant morbidity and mortality in both domestic (*Felis catus*) and feral cats around the world, with prevalence greatly influenced by country, geographic area, and characteristics of the populations studied (1). Feline leukemia virus (FeLV) is an enveloped single-stranded RNA virus of the Retroviridae family; it is divided into three subgroups based on differences in envelope proteins. The main subgroup, FeLV-A, is usually involved in viral transmission between individuals in both kittens and adults. FeLV-B and C are found in animals infected with FeLV-A. FeLV-B arises from recombination in the env gene between FeLV-A and the endogenous FeLV genome, while FeLV-C originates from mutations in the env gene of FeLV-A (2). Complex interactions between the host and the virus determine the outcome of FeLV infection. Currently, three different courses of infection are distinguished, classified as progressive, regressive, and abortive (3). Abortive infection is characterized by the release of viruses not integrated into host cells as proviruses. In these rare cases, the infected animal develops a sufficient humoral and cell-mediated immune response which allows virus clearance and clinical recovery. Abortive infection can only be detected by the detection of antibodies (1, 3). On the other hand, cats with progressive infection show integration of provirus and persistence of viremia/antigenemia (p27), leading to positive results in both molecular and serological tests. In regressive infections the virus is not actively produced, although proviral DNA is integrated into host cells; therefore, regressive infected cats are positive in PCR and negative in ELISA tests (3, 4) and also show reduced life expectancy due to the development of FeLV-related diseases (5, 6). Persistently viremic cats are a source of the virus that is shed via saliva, nasal swab, urine, and feces, with transmission occurring via the oro-nasal route. Transmission is facilitated by “friendly” activities such as mutual grooming and sharing water and food. These are the main routes of infection, but the virus can also be transmitted through fights and bites. In infected cats FeLV is mainly associated with anemia, leukemia, and lymphoma (7, 8).

Feline immunodeficiency virus (FIV) belongs to the genus Lentivirus within the family Reoviridae and infects species of the families Felidae and Hyaenidae. It is the causative agent of acquired immunodeficiency syndrome (AIDS) in cats, which, like AIDS in humans, is caused by HIV (9, 10). FIV infects, with a high prevalence rate, many different feline species, such as domestic cats, lions, cougars, bobcats, Pallas cats (*Otocolobus manul*), and kodkod (*Leopardus guigna*). FIV prevalence in asymptomatic cats has been calculated to range from 1 to 14%, whereas prevalence in symptomatic cats can reach 44% (11). The prevalence of infection can vary considerably and depends on factors such as age and gender, as well as indoor or outdoor housing. CD4+ T lymphocytopenia is typical of FIV infection and results in immunodeficiency leading to opportunistic infections, increased frequency of neoplasms, or neurologic syndrome (12). Individuals infected with FIV usually transmit the disease through fighting and biting (blood-on-blood contact), but sexual and vertical transmission is also possible. FIV infection is permanent and there is no known cure or recovery (13). There are currently known five FIV isolates subtypes, designated by the letters A through E on the basis of the envelope sequence (14). Subtype A was primarily found in northern Europe and California, whereas subtype B in southern Europe and in east-central United States. Subtype C has been instead mainly identified in California and British Columbia, while subtypes D and E have been found in Argentina and Japan (14, 15).

Etiologic diagnosis for FeLV and/or FIV is usually made by point-of-care ELISA testing. These tests simultaneously detect FeLV p27 antigen and antibodies to FIV p24 protein and have the advantage that they are easy to use in clinical practice and provide an almost immediate result. Doubtful or obviously false negative results can be confirmed by PCR (16).

Several global seroprevalence studies have been conducted for FIV and FeLV, and age, gender, neutering status, access to outdoor areas, and multi-cat households have been identified as risk factors for these infections. Several studies reported prevalence values for FeLV of 2.3% in Europe (17); for FIV and FeLV between 3.6 and 3.1% in the United States and Canada (18); between 5.84 and 22.26% in Brazil (19); and between 15 and 2% in Australia (20). Both FIV and FeLV showed lower prevalence in Europe, in the United States, and in Australia, probably thanks to the early introduction of protective testing and vaccination, which were not implemented in other countries. Considering that few epidemiological investigations have been performed in Italy so far, mainly focused on northern Italy and Tuscany (20, 21), we carried out a cross-sectional study to explore the prevalence of FeLV and FIV in cats from southern Italy regions (Campania, Basilicata and Calabria). In our opinion, this was necessary due to the fact that the Italian territory extends beyond the 10th latitude, with the simultaneous presence of coastal, hilly, mountainous and urban environments. These ecosystems, spatially close to each other but very different in terms of geographical characteristics, suggest possible differences also in infectious diseases prevalence (22). Overall, the aim of the present study was to provide an overview of the prevalence of FeLV and FIV infections in different cat populations living in several regions of southern Italy and to evaluate the risk factors (year, location and lifestyle) likely related FeLV and/or FIV positivity.

## 2. Materials and methods

### 2.1. Cats

A total of 1,322 cats were investigated in a 10-year period (from 2014 to 2023). The cats (owners, shelters and stray animals) came from the following 10 provinces located in 3 regions of Southern Italy: Napoli, Avellino, Benevento, Caserta, Salerno (Campania Region), Potenza (Basilicata Region), Catanzaro, Cosenza, Reggio Calabria and Vibo Valentia (Calabria Region).

### 2.2. FIV and FeLV analysis

Blood samples were collected from each animal in a Vacutainer^®^ (Vacutainer, Becton Dickinson Italia S.p.A.) without anticoagulant and forwarded to the Zooprofilactic Experimental Institute of Southern Italy (IZSM) for routinary analysis by practicing veterinarians and veterinarians of the local authorities. Vacutainers were centrifuged immediately or kept refrigerated for a maximum of 24 h. Centrifugation was carried out at 1300–1800 × *g* for 20 min and serum samples were analyzed immediately. FIV antibodies and FeLV antigens were detected using a commercial ELISA kit (SNAP FIV/FeLV Combo Plus Test; IDEXX Laboratories, Hoofddorp, The Netherlands) according to the manufacturer’s instructions. The test can simultaneously detect FeLV viral core capsid protein antigen (p27) and antibodies to FIV matrix protein, FIV capsid protein and FIV transmembrane glycoprotein (p15, p24 and gp40). Results for each disease were interpreted visually. Sensitivity and specificity of the: test are for FeLV: 92.3% (95% confidence interval (CI): 79.7–97.3%) and 97.3% (95% CI: 95.5–98.4%); for FIV: 100% (95% IC: 93.1–100%) and 99.6% (95% CI: 98.5–99.9%) for sensitivity and specificity, respectively (23).

### 2.3. Ethical approval and risk analysis

Ethical approval was not required because blood samples were sent to our laboratories for routine analysis and therefore no live animal was used in this study. For risk factor analysis animals’ information were collected using a registration form. Only data from the complete history form were collected and considered as variables for risk analysis (year of death, province, region, and lifestyle). Age, gender, and breed data were not analyzed because they were incomplete.

### 2.4. Statistical analysis

Chi-square test analysis was performed using MedCalc Statistical Software version 16.4.3 (MedCalc Software, Ostend, Belgium^1^; 2016) to compare proportions of positivity in relation to categorical dependent variables and to determine statistical significance within each class (year, region, province and lifestyle). Variables associated with seroprevalence for FIV/FeLV were applied to binary logistic models using JMP Pro version 15.0.0 (SAS Institute Inc). *p* < 0.05 was considered significant. Significant differences between categories were quantified by calculating odds ratios (OR) and their 95% confidence intervals (95% CI).

## 3. Results

### 3.1. Overall prevalence results

**Figure 1:**
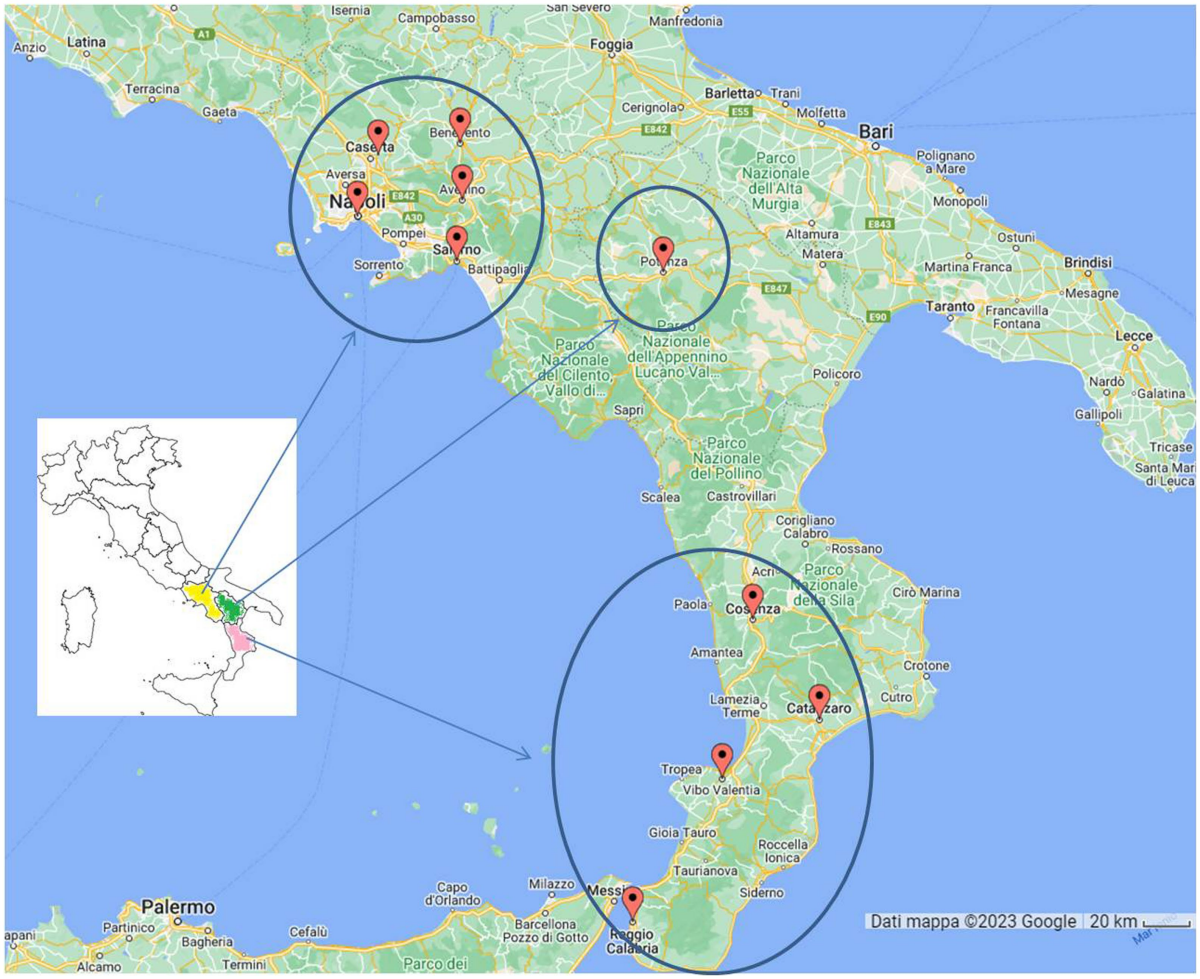
Map of cats (*n* = 1,322) sampling sites. In Campania Region (yellow colored) we analyzed 76 cats from Avellino, 166 from Benevento, 119 from Caserta, 782 from Napoli and 72 from Salerno. In Basilicata region (green colored) we analyzed 12 samples from Potenza. In Calabria region (pink colored) we analyzed 1 cat from Cosenza, 53 from Catanzaro, 39 from Reggio Calabria and 2 from Vibo Valentia.

A total of 1,322 cats living in 10 provinces of Southern Italy (Figure 1) were tested for FIV and FeLV status from 2014 to 2023. Information on sampling period, region, province and lifestyle was recorded for all samples. Gender and age were not recorded for all animals and therefore were not included in the statistical analysis. Briefly, the 1,322 serum samples were collected in ten annual periods: 2014 (16%, *n* = 210), 2015 (14% *n* = 186), 2016 (12% *n* = 158), 2017 (5%, *n* = 66), 2018 (7% *n* = 98), 2019 (9% *n* = 121), 2020 (6% *n* = 76), 2021 (12% *n* = 158), 2022 (18% *n* = 236), and 2023 (1% *n* = 13). Of the samples, 95% were from the Campania region, 7% from the Calabria region, and the rest (1%) from Basilicata. In particular, the province of Naples provided the most samples (59%, *n* = 782), followed by Benevento (13%, *n* = 166), Caserta (9%, *n* = 119), Salerno (14%, *n* = 143), Avellino (6%, *n* = 76), Catanzaro (4%, *n* = 53), Reggio Calabria (3%, *n* = 39), Potenza (1%, *n* = 12) and Vibo Valentia and Catanzaro (>0.2%). Overall, 13.99% (210/1322, 95% confidence intervals (CI): 12.12–15.86%) of the tested cats were positive for at least one of the two retroviruses (Table 1). In addition, cats simultaneously positive for FIV and FeLV were 1.97% (26/1322, 95% CI: 1.22–2.72%).

**Table 1:** Prevalence of Retroviral infection and risk factor analysis (Southern Italy, 2014–2023).

Factor	*n*	Positive	%	SE%	95% CI	*X* ^2^	*p*	OR	95% CI
Total	1,322	185	13.99	1.87	12.12–15.86				
Province									
Avellino	76	14	18.42	8.72	9.71–27.14			Ref.	
Benevento	166	36	21.69	6.27	15.42–27.96			1.1	0.4–3.3
Caserta	119	8	6.72	4.50	2.22–11.22			1.7	0.4–7.3
Napoli	782	115	14.71	2.48	12.22–17.19			0.7	0.3–1.8
Salerno	72	5	6.94	5.87	1.07–12.82	24.7	0.0009	0.4	0.1–2.0
Potenza	12	1	8.33	15.64	0.00–23.97			0.8	0.1–4.4
Cosenza	1	0	0.00	0.00	0.00			0	0
Catanzaro	53	1	1.89	3.66	0.00–5.55			0	0
Reggio Calabria	39	5	12.82	10.49	2.33–23.31			0.8	0.1–4.4
Vibo Valentia	2	0	0.00	0.00	0.00			0	0
Region									
Campania	1,215	178	14.65	1.99	12.66–16.64			Ref.	
Basilicata	12	1	8.33	15.64	0.00–23.97	6.328	0.042	0	0
Calabria	95	6	6.32	4.89	6.43			1.1	0.6–2.1
Lifestyle									
Outdoor	966	115	12.2*	2.19	10.01–14.38	5.308	0.02	1.7	1.1–2.7
Indoor	356	26	7.3	3.55	4.6–10.01				

### 3.2. FeLV prevalence of infection and risk analysis

FeLV p27 protein antigen was present in 7.64% (164/1023, 95% CI: 13.85–18.25%) of cat samples tested (Table 2). The mean prevalence values related to FeLV positivity detected in 2021 and 2022 were statistically higher (chi-square test, *p* < 0.0001) than those observed in the other years, as summarized in Table 2. Univariate analysis showed a statistical association between provinces, lifestyle and FeLV positivity (*p* = 0.0008). The province of Avellino (6.58, 95% CI 1.01–12.5) was significantly correlated with FeLV risk with odds ratios (OR) of 2.72 (95% CI 0.63–11.7), 5.0 (95% CI 0.56–43.8) and 2.53 (95% CI 0.56–43.8) % 0.28–22.5) compared to the provinces of Caserta, Salerno and Reggio Calabria (Table 2). As expected, data analysis showed that there was a statistical association between FeLV positivity and lifestyle (*p* = 0.0042). Specifically, the highest percentage of positive animals was observed in outdoor cats, which had a prevalence of 8.8% (95% CI 7.01–10.59) with a (OR) of 2.45 (95% CI 1.37–4.37) compared to domestic cats.

**Table 2:** Prevalence of FeLV infection and risk factor analysis (Southern Italy, 2014–2023).

Factor	*n*	Positive	%	SE%	95% CI	*X* ^2^	*p*	OR	95% CI
Total	1,322	101	7.64	1.43	6.21–9.07				
Province									
Avellino	76	5	6.58	5.57	1.01–12.5			Ref.	
Benevento	166	26	15.66	5.53	10.13–21.19			0.37	0.13–1.02
Caserta	119	3	2.52	2.82	0.00–5.34			2.72	0.63–11.7
Napoli	782	63	8.06	1.91	6.15–9.96			0.80	0.31–2.06
Salerno	72	1	1.39	2.7	0.00–4.09	23.081	0.0008	5.0	0.56–43.8
Potenza	12	1	8.33	15.7	0.00–23.97			0.77	0.08–7.26
Cosenza	1	0	0	0	0			0	0
Catanzaro	53	0	0	0	0			0	0
Reggio Calabria	39	2	5.13	6.92	0.00–12.05			2.53	0.28–22.5
Vibo Valentia	2	0	0	0	0			0	0
Region									
Campania	1,215	98	8.07	1.53	6.53–9.6			Ref.	
Basilicata	12	1	8.33	15.6	0.00–23.97	4.445	0.1084	0.965	0.12–7.55
Calabria	95	2	2.11	2.89	0.00–4.99			8.07	1.11–58.5
Lifestyle									
Outdoor	966	85	8.8	1.79	7.01–10.59	8.204	0.0042	2.45	1.37–4.37
Indoor	356	14	3.93	2.02	1.91–5.95				

### 3.3. FIV prevalence of infection and risk analysis

A total of 110/1322 serum samples tested (8.32%; 95% CI: 6.83–9.81) were positive for FIV antibodies (Table 3). Statistical results showed a statistical association (*p* = 0.0197) between FIV positivity detected in 2018 (9.18%; 95% IC: 3.47–14.9) and 2021 (11.39%; 95% IC: 6.44–16.35) and lifestyle (outdoor cats 9.73, 95% IC: 7.86–11.6). Notably, outdoor cats showed a higher probability of FIV seropositivity than indoor cats with an OR of 2.29 (CI 95% 1.32–3.94.

**Table 3:** Prevalence of FIV infection and risk factor analysis (Southern Italy, 2014–2023).

Factor	*n*	Positive	%	SE%	95% CI	*X* ^2^	*p*	OR	95% CI
Total	1,322	110	8.32	1.49	6.83–9.81				
Province									
Avellino	76	10	13.6	7.60	5.56–20.76			Ref.	
Benevento	166	14	8.43	4.23	4.21–12.66			1.64	(0.69–3.89)
Caserta	119	5	4.20	3.60	0.60–7.81			3.45	(1.13–10.5)
Napoli	782	72	9.21	2.03	7.18–11.23			1.49	(0.73–3.03)
Salerno	72	4	5.56	5.29	0.26–10.85	10.94	0.281	2.57	(0.76–8.62)
Potenza	12	0	0.00	0.00	0.00			0.00	0.00
Cosenza	1	0	0.00	0.00	0.00			0.00	0.00
Catanzaro	53	1	1.89	3.66	0.00–5.55			7.87	(0.97–63.5)
Reggio Calabria	39	4	10.26	9.52	0.73–19.78			1.32	(0.38–4.53)
Vibo Valentia	2	0	0.00	0.00	0.00			0.00	0.00
Region									
Campania	1,215	105	8.64	1.58	7.06–10.22				
Basilicata	12	0	0.00	0.00	0.00–0.00	2.418	0.2985	1.70	0.67–4.28
Calabria	95	5	5.26	4.49	0.77–9.75				
Lifestyle									
Outdoor	966	94	9.73	1.87	7.86–11.6	8.67	0.0032	2.29	1.32–3.94
Indoor	356	16	4.49	2.15	2.34–6.65				

**Figure 2:**
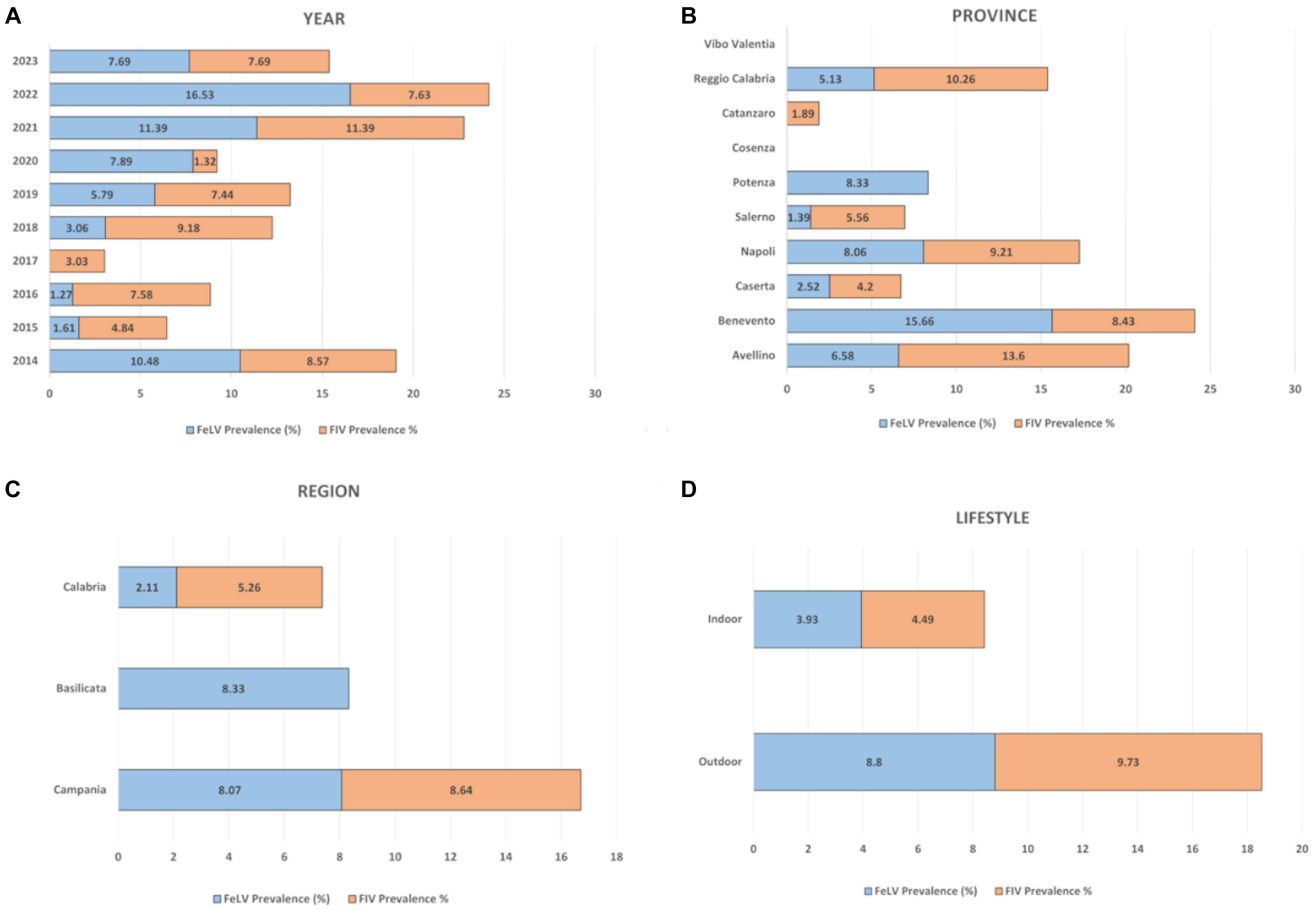
Overall percentage distribution of FIV and FeLV according to the variables considered. **(A)** year, **(B)** province, **(C)** region, **(D)** lifestyle.

No statistical significance was found between FIV seropositivity and location (region and province) (Table 3). Results are summarized in Figure 2.

## 4. Discussion

This is one of the largest cross sectional studies on FeLV and FIV cat infections conducted in Italy and particularly in southern Italy. In our research, results showed that approximately 14% of the cats tested were positive for at least one retrovirus (185/1322; 95% CI: 12.12–15.86). Furthermore, the individual prevalence of the two viral infections roughly overlapped, we indeed found a prevalence of 7.64% (95% CI: 6.21–9.07) and of 8.32% for FeLV and FIV, respectively.

As a matter of fact, prevalence of these retroviral diseases is strongly influenced by the country and/or by the region and/or by the characteristics of the population analyzed and studies conducted in different countries of the world have shown variable, not very high prevalence values for both the diseases (1, 5, 16, 18, 19, 24–27).

With respect to FeLV infection in the world, in literature prevalence values registered in Europe were between 0 and 15.6% (5), ranged from 2.3 to 3.3% in North America (16, 18, 28), were around 22.3% in Latin America (19), and from 0 to 2.9% in Asia (29–31). In addition, an overall prevalence of FeLV infection of 2.3% was found in a prospective study involving 32 European countries (17). Comparing FeLV prevalence found in this study (7.64%) with those in the other countries we observed a higher value with respect to positivity percentages described in Finland (1%) (32), Belgium (0.7%) (27), Germany (1.8%) (26), Ireland (3.3%), United Kingdom (2.3%) (7, 33) and Turkey (3.3%) (34). Prevalence values like ours were instead described by Rypuła et al. (35) in a study conducted in Poland in 2010 (6.4%). On the other hand, FeLV showed higher prevalence with respect to us in Hungary and Lebanon with values of 11.8 and 13.84%, respectively (10, 24). Furthermore, our values were in line with those registered in the few epidemiological studies performed in Italy, namely between 3.8% described in northern Italy (20) and 8.4% found in the Tuscany region (central Italy) (21).

According to our results, the probability of FeLV positivity was mainly related to the year, location (province), and lifestyle, but not to the regional origin of the tested cat. Indeed, animals living in the provinces of Caserta and Reggio Calabria had a higher probability of being FeLV positive than those living in the other selected provinces (*p* < 0.0001). FeLV is transmitted both horizontally and vertically, has a worldwide distribution, and it is known that the prevalence of the disease is influenced by the lifestyle of the animals. Indeed, as expected, cats living outdoor were more likely to be disease-positive (*p* = 0.0042) than cat living indoor cats, with an OR of 2.45.

The low prevalence of FeLV infection in our study may be related to factors not exclusively associated to the virus or to host biology, such as the particular husbandry of domestic cats. These factors should be further investigated to identify other potential risk factors associated with FeLV infection that are currently unknown or underestimated.

Interesting results were retrieved also from FIV data obtained in our cross-sectional study. FIV is an important viral infection primarily occurring through parenteral inoculation of viral particles present in the saliva or blood of infected animals (15) and, unfortunately, sick cats cannot be cured. Infection occurs throughout the world at different prevalences: 3.6–6.4% in North America (18, 36), 5.8% in South America (19), 1.5–20.1% in Asia (25, 29–31).

When comparing prevalence found in our study (8.32%) (CI 95%: 6.83–9.81) with those described in literature, we observed lower values with respect to Greece (9.2%) (5), Belgium (18.8%) (27), United Kingdom (9.5%) (7), Ireland (10.4%) (33), Hungary (9.9%) (10), Turkey (22.3%) (34), and Lebanon (18.84%) (24). In contrast, lower prevalence values were found in Finland (6.6%) (32) and Poland (4.3%) (35). Again, as already observed for FeLV, our FIV prevalence value (8.32%) was aligned with those registered in previous surveys in our country as being in between the 6.6% of northern Italy and the 11.3% of central Italy (Tuscany) (20, 21). With respect to risk analysis, data showed that there was no statistical association between FIV positivity and place of residence (province: *p* = 0.281; region: *p* = 0.2985), whereas the risk of FIV positivity was significantly correlated with year of diagnosis (*p* = 0.0197) and lifestyle. Indeed, cats living outdoors were more likely to be positive for FIV (*p* = 0.0032) than cats living indoors, with an OR of 2.29, like observed also for FeLV.

The results have shown that the prevalence of FIV remained almost constant in recent years. Therefore, it would be necessary to implement information programs for owners and to carry out anti-FIV education programs for the personnel in charge. We must not forget that there is no vaccination against FIV in Italy. Therefore, the management of positive cats and health prevention are the only measures that could to help reduce the incidence of the disease associated with FIV.

When referring to coinfection, low prevalence values were observed in our study (1.97, 95% CI: 1.22–2.72), which is consistent with data from the literature. Very low prevalence values were indeed described in Ireland (0.55%) (33), in the United Kingdom (0.42%) (7), and in Hungary (0.03%) (10). Coinfection rates like those from our study were found in Lebanon (1.46%), Thailand (2.7%), and Malaysia (2.6%) (24, 30, 31). Low rates were also found in Italy, where coinfection was 0.98% in Tuscany and 1% in northern Italy (20, 21).

However, caution should be exercised when comparing results obtained in different geographic areas, with different serologic tests, and with different sampling methods, because all of these variables may influence the prevalence value and overestimate or underestimate the impact of infectious disease on the target population. A strength of our work is that in almost all the studies cited above, as in our study, the prevalence values of FeLV and FIV were obtained by point-of-care testing (the combination SNAP was found most frequently). Therefore, the values obtained in our study can be compared with those obtained in similar researches as those mentioned above. On the other hand, some limitations should be noted when interpreting the data from our study. For example, we did not carry out any confirmation assay such as Western blot for FIV and immunofluorescence antibody test for FeLV diagnosis (24) on our positive results (obtained by SNAP test).

In any case, since the FIV test used in this study had good performance, with sensitivity, specificity, positive predictive value (PPV), and negative predictive value (NPV) of 100, 99.6, 94.5 and 100%, respectively (23), the risk of false positive and false negative results should be minimal. In contrast, the FeLV test we used had a sensitivity, specificity, PPV, and NPV of 92.3, 97.3, 73.5, and 99.4%, respectively (23), and therefore could yield false positive and false negative results. In addition, we should note that recently infected cats may have serum levels of FIV-specific antibody or FeLV p27 antigen that are below the detection limit of the test, resulting in a false negative result and a reduction in the prevalence value. Furthermore, regressive FeLV infections cannot be detected without the use of molecular tests such as PCR and are therefore not included in the reported prevalence value (24), thus leading to possible underestimation of the real disease prevalence.

To our knowledge, this is the first report investigating FIV and FeLV prevalence in the cat population of southern Italy. Our study confirms the presence, at quite low, but not neglectable prevalence of FIV and FeLV also in the south of the Italian country thus highlighting the need to adopt control measures to limit their spread and at the same time to promote prevention and management practices for animal health protection. The seroprevalence rates for FeLV and FIV in Italy in the present study were similar to those previously reported in Italy (20, 21, 37). It would not be possible to draw conclusions about the stability of seroprevalence rates over time without conducting further sampling to determine whether there is a trend beyond random fluctuations. However, the results of the present study suggest that seroprevalence rates for these preventable infections have not declined at all. A concerted effort by organized veterinary medicine to improve education and awareness is needed to address the lack of progress noted in this study. Together with the results of previous studies, these findings suggest that veterinarians and shelter managers need to better adhere to existing guidelines for managing FeLV and FIV. These include testing for all owned cats, new testing for cats that have contracted the disease or may have been exposed to infected cats, vaccination against FeLV for all kittens and adult cats at risk of exposure, segregation of infected cats, and neutering of unowned free-roaming cats.

## Data availability statement

The raw data supporting the conclusions of this article will be made available by the authors, without undue reservation.

## Ethics statement

Ethical approval was not required because blood samples were sent to our laboratories for routine analysis and therefore no live animal was used in this study.

## Author contributions

GF: Conceptualization, Funding acquisition, Project administration, Resources, Supervision, Investigation, Writing – review & editing. LM: Formal analysis, Methodology, Investigation, Writing – review & editing. AP: Formal analysis, Methodology, Investigation, Writing – review & editing. ML: Formal analysis, Methodology, Investigation, Writing – review & editing. GF: Data curation, Writing – review & editing. EC: Funding acquisition, Project administration, Supervision, Writing – review & editing. MGA: Conceptualization, Data curation, Investigation, Methodology, Supervision, Validation, Writing – review & editing, Writing – original draft. SM: Conceptualization, Data curation, Methodology, Writing – original draft, Writing – review & editing.

## Footnotes

^1^ www.medcalc.org
